# Can Aliskiren be Considered as a New Novel Drug for Hypertension?

**DOI:** 10.7759/cureus.375

**Published:** 2015-11-04

**Authors:** Adnan Bashir Bhatti, Zarine Anwar Gazali

**Affiliations:** 1 Department of Medicine, Capital Development Authority Hospital, Islamabad, Pakistan; 2 Research Fellow, MITR Hospital, Kharghar, Navi Mumbai, Maharashtra, India

**Keywords:** aliskerin, hypertension, angiotensin converting enzyme, angiotensin receptor blockade

## Abstract

Hypertension is one of the most common causes of death across the globe. Many trials and drugs have been used for controlling the debilitating effects of hypertension. One such new class of drug is direct renin inhibitors (DRI), e.g., aliskiren, which block the renin-angiotensin system (RAS). It blocks the very first step in the RAS system. Multiple trials have been carried out debating the outcome of monotherapy and combination therapy with other classes of hypertensive drugs. Focus on compliance, adverse effects, and the cost have also been in the news. Extensive studies are still needed to justify the clinical use of a DRI in the effective treatment of hypertension.

## Introduction and background

**Figure 1:**
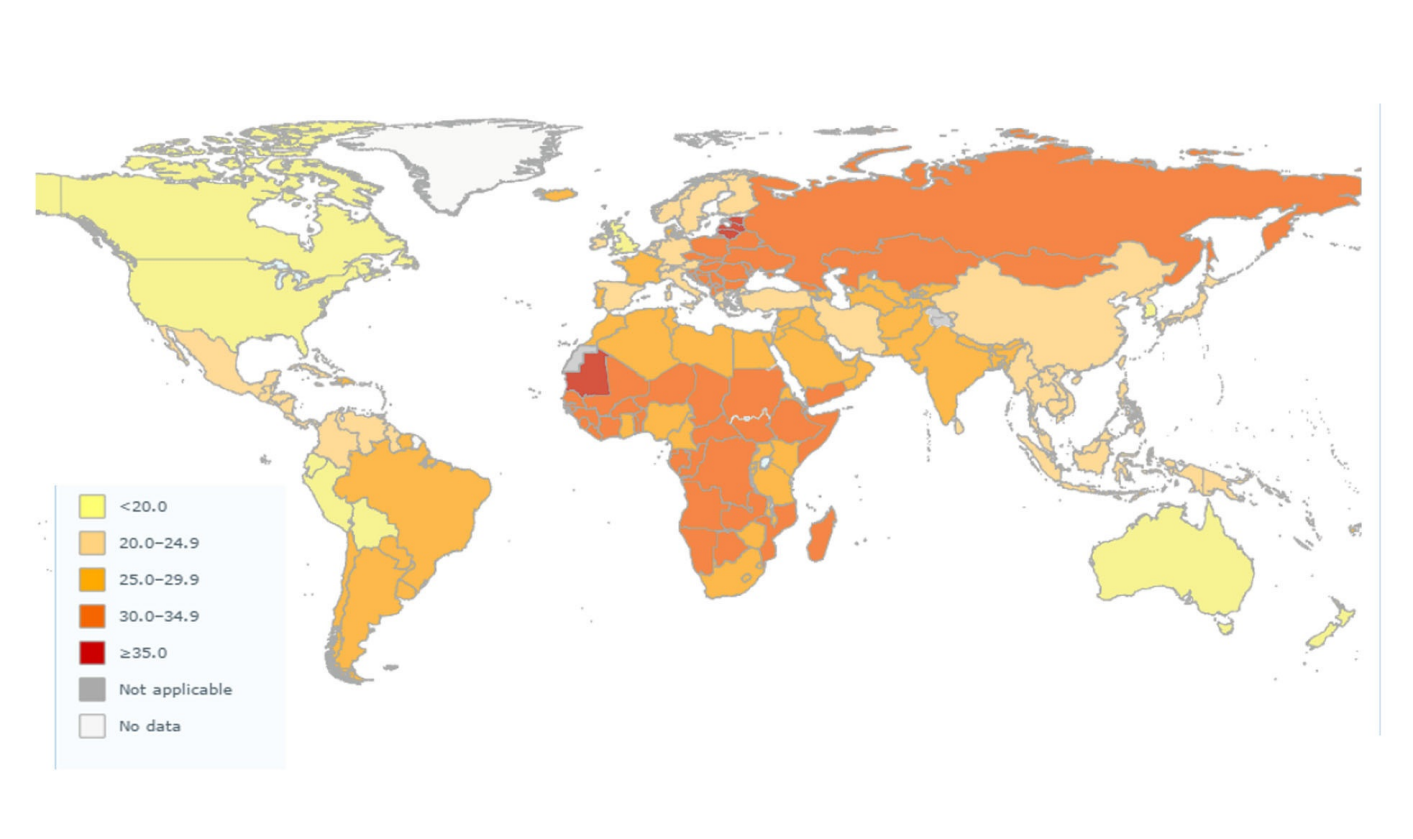
Prevalence of raised blood pressure, ages 18+ 2013-2014, figure adapted from WHO website Retrieved from WHO website: http://www.who.int/gho/ncd/risk_factors/blood_pressure_prevalence/en/

Hypertension is one among the leading factors that contribute towards the cardiovascular disease reported worldwide. It is defined as a condition in which the mean systolic blood pressure (SBP) is ≥ 140 mm of Hg and a mean diastolic blood pressure is ≥ 90 mm of Hg. According to the World Health Organisation (WHO), the overall prevalence of raised blood pressure in adults over 18 years of age was approximately 22% in 2014. The incidence of raised blood pressure is highest in Africa, where it is about 30% for both sexes. The lowest prevalence of hypertension is in America, where it is reported in about 18% of both sexes. Men (21%) in this region have a higher incidence than women do (16%). Men have a slightly higher prevalence of hypertension globally. The global prevalence and age distribution of hypertension is as represented in Figure 1 and Table 1.

**Table 1:** Age distribution of hypertension in WHO regions (Age group 18+ years). Table adapted from WHO website Retrieved from WHO website: http://apps.who.int/gho/data/view.main.2540?lang=en

WHO Region	Year	Both Sexes	Female	Male
Africa	2014	29.6 [25.8-33.2]	29.5 [24.6-34.8]	29.7 [24.5-35.6]
Africa	2010	29.7 [27-32.7]	29.6 [25.9-33.5]	29.9 [25.9-34.3]
Americas	2014	18.2 [15.6-21]	15.6 [12.4-19.3]	20.8 [16.7-25.3]
Americas	2010	19.3 [17.4-21.3]	16.8 [14.3-19.4]	22 [18.9-25.2]
South-East Asia	2014	24.7 [20.4-28.9]	24.2 [18.6-30.3]	25.3 [19.3-31.9]
South-East Asia	2010	25.1 [22.3-28.2]	24.6 [20.5-28.9]	25.6 [21.1-30.4]
Europe	2014	23.3 [20.7-26]	19.7 [16.2-23.4]	27.1 [23.2-31.3]
Europe	2010	25.1 [22.8-27.3]	21.4 [18.7-24.4]	29.1 [26-32.2]
Eastern Mediterranean	2014	26.9 [23-30.8]	26.4 [21.6-31.4]	27.5 [22.4-32.9]
Eastern Mediterranean	2010	27.6 [24.8-30.3]	27.2 [23.5-30.8]	28 [24.2-32]
Western Pacific	2014	18.7 [14.4-22.8]	16.7 [12.1-22.7]	20.6 [15.1-27.6]
Western Pacific	2010	20 [17.3-22.8]	17.9 [14.4-21.9]	21.9 [17.8-26.6]
Global	2014	22.2 [19.9-24.5]	20.5 [17.7-23.4]	24 [20.8-27.4]
Global	2010	23.2 [21.4-24.8]	21.4 [19.3-23.5]	25 [22.6-27.4]

The following data, compiled from the fact sheet published by the World Hypertension League and the International Society of Hypertension, sheds light on the current global status of hypertension. High blood pressure is responsible for about 9.4 million deaths and an estimate of 162 million years of life were lost in 2010 itself [1-8]. It is a leading risk for fetal and maternal death in pregnancy, renal failure, and dementia. Approximately four in 10 adults over 25 years of age have hypertension, and in some countries, one in five is reported to have pre-hypertension. An estimated nine to 10 individuals living to 80 years of age will develop hypertension at some point of their life. Economically developing countries carry the highest burden of hypertensive patients. Hypertension has a major economic impact on health care spending [9]. This condition has become so widespread that approximately 10% of health care expenditures are directly related to hypertension and its associated complications. An unhealthy diet is estimated to be related to about half of hypertension cases. About 30% of hypertension cases can be attributed to increased salt intake and 20% to low dietary potassium owning to the lack of fruit and vegetables in the diet. Physical inactivity and obesity are related to about 20% and 30% of hypertension, respectively. Clinical interventions are not optimal for hypertension and most individuals with hypertension are unaware about their condition. A significant number of patients who are aware of their hypertension remain untreated, and even when treated, a large proportion still has sub-optimally controlled blood pressure [10-11].

## Review

Over the years, many strategies and drug approaches have been used to reduce the hazardous effects of hypertension. The drug, aliskerin, is a comparatively new drug available for the treatment of hypertension. This paper shall thus review the safety, efficacy, and its use alone or in combination with other hypertensive drugs [12].

### Pharmacology of aliskiren

**Figure 2:**
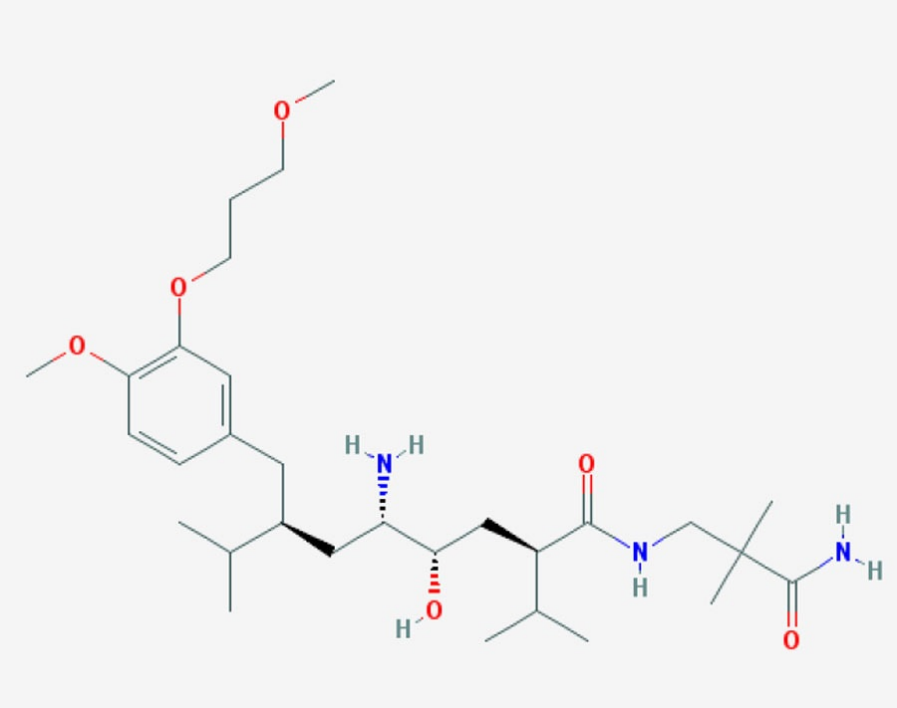
Chemical structure of Aliskiren Structure retrieved from NCBI site : http://pubchem.ncbi.nlm.nih.gov/compound/Aliskiren#section=Top

Aliskiren (2S,4S,5S,7S)-N-(2-carbamoyl-2-methylpropyl)-5-amino-4-hydroxy- 2,7-diisopropyl-8-[4-methoxy-3-(3-methoxypropoxy-)phenyl)-octanamide was approved for the first time as a drug for hypertension by the US food and Drug Administration in March 2007. The structure of the compound is as given below (Figure 2). It is the first orally effective DRI approved for the treatment of hypertension. It is a non-peptide molecule and has a low molecular weight. As it a non-peptide molecule, it has better bioavailability and a long half-life and can, therefore, lower blood pressure effectively [13]. It is available in the form of 150 mg and 300 mg doses. Once administered orally, the effect of the drug peaks in one to three hours, achieves its steady state in five to seven days, and has a half-life of 40 hours [14].

### Mechanism of action of DRI

**Figure 3:**
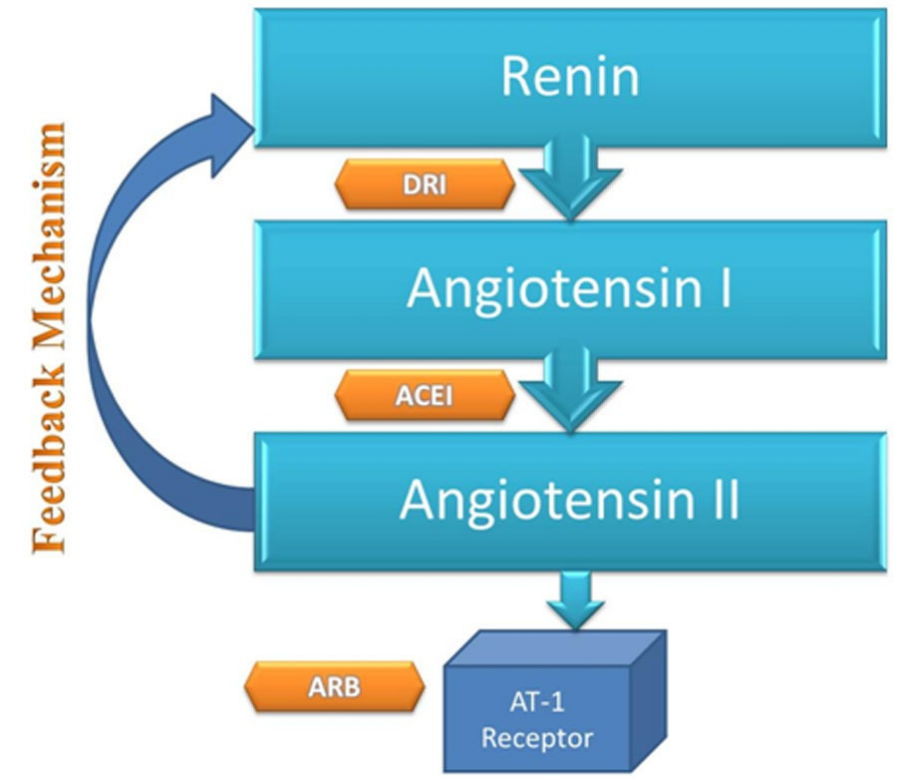
Action sites of DRI, ACEI and ARB

DRIs are known to inhibit the RAS and can reduce cardiovascular risk. Their main aim is to prevent the formation of angiotensin I by blocking renin from converting to angiotensin I [15-16]. It is the hydrolysis of Leu10-Val 11 bond of angiotensinogen which leads to the generation of decapeptide fragment, angiotensin I, which is the first and the rate-limiting step of RAS. DRIs are the only drug that can act on this pathway and are unique in its own sense [17]. Drugs like angiotensin-converting enzyme inhibitors (ACEI) and angiotensin receptor blockers (ARB) act on angiotensin II and AT 1 receptors, respectively (Figure 3) [18-19].

### Aliskiren as monotherapy

When a dose of 150 mg of aliskiren was administered, SBP was found to be reduced by 10-15 mm of Hg and DBP by 2-10 mm of Hg. With doses of 300 mg, SBP showed a further reduction of 12-16 mm of Hg and DBP by 5-11 mm of Hg. However, all the studies conducted so far have tested it with other hypertensive drugs and have concluded that it could be only used as an additional drug for hypertension [20]. No effect was seen in doses less than 75 mg or more than 300 mg. DRI is reported to take about two weeks to bring out the observational changes in blood pressure and their effect can be best observed by four weeks [21-25]. In a recent study conducted by a group of researchers to check the efficacy of aliskiren, the mean SBP was reported as 5.9, 12.5, and 15.2 mm of Hg with placebo, aliskiren (150 mg/day), and aliskiren (300 mg /day) and DBP as 6.2, 10.1, and 11.8, respectively [26]. Unlike many other hypertensive drugs, DRI does not increase the plasma renin activity.

### Diabetic kidney disease

Diabetic kidney disease (DKD) has been the leading cause of end-stage renal disease [27]. Drugs such as ACEI and ARB have been the basis of management in DKD. Patients with DKD present with progressive renal injury [28]. This occurs due to the activation of the renin-angiotensin-aldosterone system (RAAS), which in turn stimulates the AT1 receptors, thus increasing the production of angiotensin II that causes podocyte injury along with damage to the basement membrane and glomerular epithelium [29-30]. ACEI are known to block the conversion of angiotensin I to angiotensin II, thereby reducing the concentration of angiotensin II in the system. This would in turn result in the loss of feedback inhibition, hence, causing an increase in renin release. This leads to renal damage [31]. ARBs act on AT1 receptors, which interrupt the negative feedback control of renin release and cause an increase in plasma renin activity, along with an increase in angiotensin I and angiotensin II. This leads to inflammation, fibrosis, and oxidant injury of the organs, including the kidneys [32-33].

### Is aliskiren superior to ARBS and ACEI in DKD?

On administration of aliskiren, a reduction in levels of proteinuria and hypertension have been noted. The use of aliskiren has been shown to decrease tubulointerstitial fibrosis, oxidative markers, glomerular pressure, and podocytopathy [34-35].

In a randomized, double-blinded, placebo-controlled, multinational study, the use of aliskiren for 24 weeks showed a 20% reduction in albuminuria with small differences in blood pressures. Five hundred and ninety-nine hypertensive patients with Type 2 diabetes and nephropathy on treatment with losartan (100 mg per day) were randomly selected for aliskiren treatment or placebo. The study concluded that aliskiren does have an additional renoprotective effect with mild lowering of blood pressure properties when used in combination to ARBs [36].

Another similar study conducted to learn about the effectiveness of dual therapy over monotherapy in the management of the cardiovascular and renal system was stopped due to adverse effects, including hypotension, hyperkalemia, and acute renal injury, with the use of ACEI and ARB [37]. This study has led to a debate ever since.

The Aliskiren Trial on Acute Heart Failure Outcomes (ASTRONAUT) was conducted to study the effects of DRI with ACEI/ARB vs placebo with ACEI/ARB in hypertension. The result indicates that there is no significant difference in cardiovascular parameters under DRI treatment [38]. The summary and outcome of all the above-mentioned trials is as shown in Table 2. 

**Table 2:** Summary of trials involving aliskiren

Clinical Trial	Type of Trial	Drugs & Dose	Comments	References
AVOID trial	Randomized, double blinded, placebo-controlled	Aliskiren (150mg to 300mg) OD after 3 months + losartan (100mg) OD	Aliskerin is renoprotective. (20% reduction in albuminuria and 50% reduction in urinary albumin:creatine ratio (UACR)	[36]
ATTITUDE	A multicentre Randomized double-blinded placebo-controlled trial	Aliskiren300 mg vs placebo OD for 4 years along with ACEI and ARB	Increased adverse effects with no significant difference.	[37]
ASTRONAUT	Clinical trial	DRI with ACEI/ARB vs placebo with ACEI/ARB	Adverse effects lesser than those seen in ATTITUDE with no significant difference	[38]

### Debate on aliskiren with other ACEI and ARB

A recent study published in the European Heart Journal carried out by Tea, et al. focuses on the use of aliskiren as monotherapy or in combination with other hypertensive drugs. The study was a randomised placebo-controlled clinical trial, including aliskiren (300 mg) and hydrochlorothiazide (HCTZ) (25 mg) or amlodipine (5 mg). The group that had received aliskiren treatment showed a significant improvement with 35% of them reporting an average SBP of < 130 mm Hg and 11% of them with < 120 mm Hg as compared to 22.8% and 6.8% in the placebo groups [39].

Similarly, in another study named the ONTARGET (Ongoing Telmisartan Alone and in Combination with Ramipril Global Endpoint Trial), the patients who were treated with DRI and ramipril/telmisartan as combination therapy led to a reduction of 2-3 mm of Hg in SBP when compared to that of those who received monotherapy. However, no benefit was observed in cases of stroke, myocardial infarction, or hospital stay for heart failure [40-41]. 

As per the research conducted by Oparil and co-workers, aliskiren and valsartan were found to be better when given in combination as the dual blockage of RAAS reduces the blood pressure more effectively [42].

### Take on DRI with calcium channel blockers (CCB) and diuretics

The ACCELERATE (Aliskiren and the Calcium-Channel Blocker Amlodipine Combination as an Initial Treatment Strategy for Hypertension) trial was a single-blinded placebo run with three consecutive phases of double-blind active treatment. Out of 315 patients enrolled in the study; 50% of the patients were treated with DRI (150 mg) while the other 50% were treated with amlodipine (5 mg) monotherapy during Phase I up to 16 weeks. Phase II was from 16 to 24 weeks where the patients received a combination of the drugs; in Phase III, a few patients received hydrochlorothiazide or placebo depending upon their BP. The SBP and DBP were measured at 8, 16, and 24 weeks, which showed a decrease of 6.5 mm of Hg in monotherapy and 1.4 mm of Hg in combination. The results were promising with DRI until Phase II; it was highly recommended to use a combination of both the drugs to reduce the BP rather than as a monotherapy [43].

Another study called The HOPE (Heart Outcomes Prevention Evaluation) trial concluded that ramipril can significantly reduce the cardiovascular morbidity and mortality in people suffering from heart diseases [44]. Andersen, et al. carried out a major study comprised of 842 patients to compare the effectiveness of DRI and CCB. In his study, he considered aliskiren (DRI) (150 mg titrated to 300 mg at week six; n = 420) and ramipril (CCB) (5 mg titrated to 10 mg at week six; n = 422). Hydrochlorothiazide was added at week 12 (12.5 mg, titrated to 25 mg, if required). Only 697 patients completed the trial. It was observed that SBP decreased significantly with DRI as opposed to ramipril, i.e., 16.9 vs 13.2 mm of Hg, respectively. The difference in the DBP was at 13.3 vs 11.6 mm Hg. Aliskiren-based therapy also provided high BP control over CCB or diuretics [45].

### Overweight individuals and DRI impact

The (Aliskiren in Left Ventricular Hypertrophy) ALLAY trial, registered obese patients (body mass index > 25 kg/m^2^) who were suffering from hypertension and left ventricular hypertrophy (LVH) to undergo treatment with DRI or ARB or both. The DRI in consideration was aliskiren and ARB was losartan. The trial showed that DRI can effectively reduce the LVH, which is an important sign of end organ damage and, hence, is a safe drug. Controversies following the ATTITUDE trial crept in, questioning the safety of the drug, but this trial proved that there was no difference in adverse effects in both the groups [47].

### DRI and heart failure

The ALOFT (Aliskiren Observation of Heart Failure Treatment) trial shed light on the potential benefit of using DRI in patients with heart failure. The study was a randomised controlled trial considering beta blockers, ACEI, and DRI. The result revealed that there was a significant reduction in brain natriuretic peptide (BNP) concentration in groups receiving DRI [48]. The addition of aliskiren was found to have a positive effect on neurohumoral dynamics of heart failure and was found to be well tolerated by the patients.

### Ongoing trials

The ATMOSPHERE trial is being conducted to determine the safety and efficacy of aliskiren and enalapril combination on morbi-mortality in patients with congestive heart failure. The APOLLO (A Randomized Controlled Trial of Aliskiren in the Prevention of Major CV Events in Elderly People) trial aims to determine the action of aliskiren alone and in combination with HCTZ or amlodipine in patients above 65 years. The AQUARIUS (Safety and Efficacy of Aliskiren on the Progression of Atherosclerosis in Coronary Artery Disease Patients) intends to study the change in coronary atherosclerotic disease through aliskiren treatment [49].

### DRI and metabolic syndrome

Aliskiren is capable of conferring increased insulin sensitivity, both in humans and animals, after a high fructose diet. Aliskiren achieved this via effectively decreasing the angiotensin II formation. Male Sprague-Dawley rats demonstrating hypertension, hyperinsulinaemia, insulin resistance, hyperglycemia, hypercholesterolemia, and hypertriglyceridaemia after a high-fructose diet were shown to resume normalcy by the administration of subcutaneous aliskiren (100 mg/kg/daily). Obese mice that were supplemented with a fat-rich diet showed a reduction in plasma leptin levels and insulin resistance post-administration of aliskiren [50-52].

### Which is better in co-morbid patients (Type 2 diabetes with hypertension) - DRI or calcium channel blocker/angiotensin-receptor blocker?

A recently published work by a group of researchers has proved to be an eye-opener for diabetic patients suffering from hypertension. The study revolved around the efficacy and safety of DRI against CCB/ARB in hypertensive patients with Type 2 diabetes mellitus. It was comprised of 126 patients who were treated for 24 weeks with aliskiren and CCB/ARB. The results revealed a significant reduction in SBP and DBP with DRI, i.e, 11.37% and 10.67%, respectively, while patients with CCB/ARB showed a reduction of 8.47% and 9.28%, respectively. DRI also showed a significant reduction in microalbuminuria as compared to CCB/ARB after a period of six months. Hence, the addition of aliskiren to regular hypertensive drugs can be highly effective in reducing the blood pressure and urinary albumin excretion [53-57].

### Compliance and persistence better with DRI

An original cohort study discussing the drug costs and compliance of DRI with ACEI and ARB have been published recently. The study had included 17,902 patients out of which 1,395 patients were on aliskiren. Aliskiren was used in conjunction with valsartan (n = 497, 35.6%) in one cohort, and in the other, valsartan was used with lisinopril (n = 3447, 20.9%). The mean age in the aliskiren group was 66.4 and that of the ACEI with ARB group was 64.5 years. About 42.7% were males and 51.6% were women. Patients on DRI with ARB demonstrated less discontinuation rate, better compliance, and less hospitalisation when compared to that of ACEI and ARB [58]. In an open-label, multicenter study, patients aged between 65-74 years responded well to the drug, and therefore, it as suggested that DRI does not require any dose adjustments [59].

### Treatment expenditure

A study focused on determining the overall medical services cost per patient with hypertension found out that DRI, plus ARB treatment, requires $1,584 and ACEI and ARB requires only $734. The cost difference was $850. The exclusive hypertension-related medical services cost averages around $1,292 per patient for DRI and ARB, in contrast to $656 with ACEI and ARB [49]. The study proved that patients using DRI with ARB would have to bear more cost than the classical ACEI and ARB. However, since the study was retrospective in nature, an exact correlation cannot be made [50]. To date, thiazide diuretics are preferred for treating of hypertension due to it being the most economical drug. Aliskiren (150 mg) once daily for a 28 day cycle costs $31.06 US while losartan (50 mg) is at $20.08 US, irbesartan (150 mg) $19.72 US, and amlodipine (5 mg) once daily at $2.01 US [60-61].

## Conclusions

It can thus be concluded the prospective of using aliskiren as a combination therapy along with a conventional hypertensive drug is highly promising. However more research and clinical trials are required to carefully devise a safe and effective treatment course. Furthermore, owing to the relative higher cost to DRI drugs when compared to other conventional drugs, research is also warranted in a direction to reduce the cost of DRI drugs under study so that patients can benefit from the advantages of using them without a significant economic burden.

## References

[1] Lim SS, Vos T, Flaxman AD (2012). A comparative risk assessment of burden of disease and injury attributable to 67 risk factors and risk factor clusters in 21 regions, 1990-2010: a systematic analysis for the Global Burden of Disease Study 2010. Lancet.

[2] World Health Organization (WHO) (2015). A Global Brief on Hypertension: Silent Killer, Global Public Health Crisis. World Health Day, World Health Organization.

[3] Levy D, Larson MG, Vasan RS, Kannel WB, Ho KK (1996). The progression from hypertension to congestive heart failure. JAMA.

[4] Udani S, Lazich I, Bakris Bakris (2011). Epidemiology of hypertensive kidney disease. Nat Rev Nephrol.

[5] Levi Marpillat N, Macquin-Mavier I, Tropeano AI, Bachoud-Levi AC, Maison P (2013). Antihypertensive classes, cognitive decline and incidence of dementia: a network meta-analysis. J Hypertens.

[6] Khan KS, Wojdyla D, Say L, Gülmezoglu AM, Van Look PF (2006). WHO analysis of causes of maternal death: A systematic review. Lancet.

[7] Seely EW, Maxwell C (2007). Cardiology patient page: Chronic hypertension in pregnancy. Circulation.

[8] Chen G, McAlister FA, Walker RL, Hemmelgarn BR, Campbell NR (2011). Cardiovascular outcomes in Framingham participants with diabetes: The importance of blood pressure. Hypertension.

[9] Gaziano TA, Bitton A, Anand S, Weinstein MC; International Society of Hypertension (2009). The global cost of nonoptimal blood pressure. J Hypertens.

[10] Perkovic V, Huxley R, Wu Y, Prabhakaran D, MacMahon S (2007). The burden of blood pressure-related disease: A neglected priority for global health. Hypertension.

[11] Ibrahim MM, Damasceno A (2012). Hypertension in developing countries. Lancet.

[12] Go AS, Mozaffarian D, Roger VL, Benjamin EJ, Berry JD, Borden WB, Bravata DM, Dai S, Ford ES, Fox CS, Franco S, Fullerton HJ, Gillespie C, Hailpern SM, Heit JA, Howard VJ, Huffman MD, Kissela BM, Kittner SJ, Lackland DT, Lichtman JH, Lisabeth LD, Magid D, Marcus GM, Marelli A, Matchar DB, McGuire DK, Mohler ER, Moy CS, Mussolino ME, Nichol G, Paynter NP, Schreiner PJ, Sorlie PD, Stein J, Turan TN, Virani SS, Wong ND, Woo D, Turner MB; American Heart Association Statistics Committee and Stroke Statistics Subcommittee (2013). Heart disease and stroke statistics—2013 update: a report from the American Heart Association. Circulation.

[13] Wood JM, Maibaum J, Rahuel J, Grütter MG, Cohen NC, Rasetti V, Rüger H, Göschke R, Stutz S, Fuhrer W, Schilling W, Rigollier P, Yamaguchi Y, Cumin F, Baum HP, Schnell CR, Herold P, Mah R, Jensen C, O'Brien E, Stanton A, Bedigian MP (2003). Structure-based design of aliskiren, a novel orally effective renin inhibitor. Biochem Biophys Res Commun.

[14] Nussberger J, Wuerzner G, Jensen C, Brunner HR (2002). Angiotensin II suppression in humans by the orally active renin inhibitor Aliskiren (SPP100): comparison with enalapril. Hypertension.

[15] Jacoby DS, Rader DJ (2003). Renin-angiotensin system and atherothrombotic disease: from genes to treatment. Arch Intern Med.

[16] Schmidt-Ott KM, Kagiyama S, Phillips MI (2000). The multiple actions of angiotensin II in atherosclerosis. Regul Pept.

[17] James MN, Sielecki AR (1985). Stereochemical analysis of peptide bond hydrolysis catalyzed by the aspartic proteinase penicillopepsin. Biochemistry.

[18] Verdecchia P, Angeli F, Mazzotta G, Gentile G, Reboldi G (2008). The renin angiotensin system in the development of cardiovascular disease: role of aliskiren in risk reduction. Vasc Health Risk Manag.

[19] Schweda F, Kurtz A (2012). Regulation of renin release by local and systemic factors. Reviews of Physiology, Biochemistry and Pharmacology.

[20] Oparil S, Yarows SA, Patel S, Fang H, Zhang J, Satlin A (2007). Efficacy and safety of combined use of aliskiren and valsartan in patients with hypertension: a randomized, double-blind trial. Lancet.

[21] Kushiro T, Itakura H, Abo Y, Gotou H, Terao S, Keefe DL (2006). Aliskiren, a novel oral renin inhibitor,provides dose-dependent efficacy and placebo-like tolerability in Japanese patients with hypertension. Hypertens Res.

[22] Jordan J, Engeli S, Boye SW, Le Breton S, Keefe DL (2007). Direct renin inhibition with aliskiren in obese patients with arterial hypertension. Hypertension.

[23] Oh BH, Mitchell J, Herron JR, Chung J, Khan M, Keefe DL (2007). Aliskiren, an oral renin inhibitor, provides dose-dependent efficacy and sustained 24-hour blood pressure control in patients with hypertension. J Am Coll Cardiol.

[24] Pool JL, Schmieder RE, Azizi M, Aldigier JC, Januszewicz A, Zidek W, Chiang Y, Satlin A (2007). Aliskiren, an orally effective renin inhibitor, provides antihypertensive efficacy alone and in combination with valsartan. Am J Hypertens.

[25] Villamil A, Chrysant SG, Calhoun D, Schober B, Hsu H, Matrisciano-Dimichino L, Zhang J (2007). Renin inhibition with aliskiren provides additive antihypertensive efficacy when used in combination with hydrochlorothiazide. J Hypertens.

[26] Dahlöf B, Anderson D, Arora V (2007). Aliskiren a direct renin inhibitor, provides antihypertensive efficacy and excellent tolerability independent of age or gender in patients with hypertension. J Clin Hypertens.

[27] Carey RM, Siragy HM (2003). The intrarenal renin-angiotensin system and diabetic nephropathy. Trends Endocrinol Metab.

[28] Price DA, Porter LE, Gordon M, Fisher ND, De'Oliveira JM, Laffel LM, Passan DR, Williams GH, Hollenberg NK (1999). The paradox of the low-renin state in diabetic nephropathy. J Am Soc Nephrol.

[29] Hoffmann S, Podlich D, Hähnel B, Kriz W, Gretz N (2004). Angiotensin II type 1 receptor overexpression in podocytes induces glomerulosclerosis in transgenic rats. J Am Soc Nephrol.

[30] Whaley-Connell AT, Chowdhury NA, Hayden MR, Stump CS, Habibi J, Wiedmeyer CE, Gallagher PE, Tallant EA, Cooper SA, Link CD, Ferrario C, Sowers JR (2006). Oxidative stress and glomerular filtration barrier injury: Role of the renin-angiotensin system in the Ren2 transgenic rat. Am J Physiol Renal Physiol.

[31] Rashid HU, Mende C (2011). The role of direct renin inhibition in clinical practice focus on combination therapy. Am J Cardiovasc Drugs.

[32] Strauss MH, Hall AS (2006). Angiotensin receptor blockers may increase risk of myocardial infarction: unraveling the ARB-MI paradox. Circulation.

[33] Schrier RW (2010). Aldosterone ‘escape’ vs ‘breakthrough’. Nat Rev Nephrol.

[34] Whaley-Connell A, Nistala R, Habibi J, Hayden MR, Schneider RI, Johnson MS, Tilmon R, Rehmer N, Ferrario CM, Sowers JR (2010). Comparative effect of direct renin inhibition and AT1R blockade on glomerular filtration barrier injury in the transgenic Ren2 rat. Am J Physiol Renal Physiol.

[35] Vanourková Z, Kramer HJ, Husková Z, Cervenka L, Vanecková I (2010). Despite similar reduction of blood pressure and renal ANG II and ET-1 levels aliskiren but not losartan normalizes albuminuria in hypertensive Ren-2 rats. Physiol Res.

[36] Parving HH, Persson F, Lewis JB, Lewis EJ, Hollenberg NK; AVOID Study Investigators (2008). Aliskiren combined with losartan in type 2 diabetes and nephropathy. N Engl J Med.

[37] Parving HH, Brenner BM, McMurray JJ, de Zeeuw D, Haffner SM, Solomon SD, Chaturvedi N, Persson F, Desai AS, Nicolaides M, Richard A, Xiang Z, Brunel P, Pfeffer MA; ALTITUDE Investigators (2012). Cardiorenal end points in a trial of aliskiren for type 2 diabetes. N Engl J Med.

[38] Gheorghiade M, Albaghdadi M, Zannad F, Fonarow GC, Böhm M, Gimpelewicz C, Botha J, Moores S, Lewis EF, Rattunde H, Maggioni A; ASTRONAUT investigators and study coordinators (2011). Rationale and design of the multicentre, randomized, double-blind, placebo-controlled Aliskiren Trial on Acute Heart Failure Outcomes (ASTRONAUT). Eur J Heart Fail. 2011.

[39] Teo KK, Pfeffer M, Mancia G, O'Donnell M, Dagenais G, Diaz R, Dans A, Liu L, Bosch J, Joseph P, Copland I, Jung H, Pogue J, Yusuf S, Aliskiren Prevention of Later Life Outcomes Trial Investigators (2004). Aliskiren alone or with other antihypertensive in the elderly with borderline and stage 1 hypertension: the APOLLO trial. Eur Heart J.

[40] ONTARGET Investigators, Yusuf S, Teo KK, Pogue J, Dyal L, Copland I, Schumacher H, Dagenais G, Sleight P, Anderson C (2008). Telmisartan, ramipril, or both in patients at high risk for vascular events. N Engl J Med.

[41] Mann JF, Schmieder RE, McQueen M, Dyal L, Schumacher H, Pogue J, Wang X, Maggioni A, Budaj A, Chaithiraphan S, Dickstein K, Keltai M, Metsärinne K, Oto A, Parkhomenko A, Piegas LS, Svendsen TL, Teo KK, Yusuf S; ONTARGET investigators (2008). Renal outcomes with telmisartan, ramipril, or both, in people at high vascular risk (the ONTARGET study): a multicentre, randomised, double-blind, controlled trial. Lancet.

[42] Oparil S, Yarows SA, Patel S, Fang H, Zhang J, Satlin A (2007). Efficacy and safety of combined use of aliskiren and valsartan in patients with hypertension: a randomised,double-blind trial. Lancet.

[43] Brown MJ, McInnes GT, Papst CC, Zhang J, MacDonald TM (2011). Aliskiren and the calcium channel blocker amlodipine combination as an initial treatment strategy for hypertension control (ACCELERATE): a randomised, parallel-group trial. Lancet.

[44] Yusuf S, Sleight P, Pogue J, Bosch J, Davies R, Dagenais G (2000). Effects of an angiotensin-converting-enzyme inhibitor, ramipril, on cardiovascular events in high-risk patients. The Heart Outcomes Prevention Evaluation Study Investigators. N Engl J Med.

[45] Andersen K, Weinberger MH, Egan B, Constance CM, Ali MA, Jin J, Keefe DL (2008). Comparative efficacy and safety of aliskiren, an oral direct renin inhibitor,and ramipril in hypertension: a 6-month, randomized, double-blind trial. J Hypertens.

[46] Krop M, Garrelds IM, de Bruin RJ, van Gool JM, Fisher ND, Hollenberg NK, Jan Danser AH (2008). Aliskiren accumulates in renin secretory granules and binds plasma prorenin. Hypertension.

[47] Solomon SD, Appelbaum E, Manning WJ, Verma A, Berglund T, Lukashevich V, Cherif Papst C, Smith BA, Dahlöf B; Aliskiren in Left Ventricular Hypertrophy (ALLAY) Trial Investigators (2009). Effect of the direct renin inhibitor aliskiren, the angiotensin receptor blocker losartan, or both on left ventricular mass in patients with hypertension and left ventricular hypertrophy. Circula­tion.

[48] McMurray JJ, Pitt B, Latini R, Maggioni AP, Solomon SD, Keefe DL, Ford J, Verma A, Lewsey J; Aliskiren Observation of Heart Failure Treatment (ALOFT) Investigators (2008). Effects of the oral direct renin inhibitor aliskiren in patients with symptomatic heart failure. Circ Heart Fail.

[49] Sen S, Sabırlı S, Ozyiğit T, Uresin Y (2013). Aliskiren: review of efficacy and safety data with focus on past and recent clinical trials. Ther Adv Chronic Dis.

[50] Fogari R, Zoppi A, Mugellini A, Lazzari P, Derosa G (2010). Different effects of aliskiren and losartan on fibrinolysis and insulin sensitivity in hypertensive patients with metabolic syndrome. Horm Metab Res.

[51] Chou CL, Lai YH, Lin TY, Lee TJ, Fang TC (2011). Aliskiren prevents and ameliorates metabolic syndrome in fructose-fed rats. Arch Med Sci.

[52] Stucchi P, Cano V, Ruiz-Gayo M, Fernández-Alfonso MS (2009). Aliskiren reduces body-weight gain, adiposity and plasma leptin during diet-induced obesity. Br J Pharmacol.

[53] Imbalzano E, Scarpelli M, Mandraffino G, Creazzo M, Lizio G, Trapani G, Dattilo G, Dalbeni A, Tomasello C, Sardo MA, Saitta A (2014). Combination therapy with aliskiren versus ramipril or losartan added to conventional therapy in patients with type 2 diabetes mellitus, uncontrolled hypertension and micro albuminuria. J Renin Angiotensin Aldosterone Syst.

[54] Sowers J, Epstein M, Frolich E (2001). Diabetes, hypertension, and cardiovascular disease. An update. Hypertension.

[55] Volpe M, Cosentino F, Tocci G, Palano F, Paneni F (2011). Antihypertensive therapy indiabetes: the legacy effect and RAAS blockade. Curr Hypertens Rep.

[56] Wu M, Tung S, Hsu K, Lee CT (2014). Aliskiren add-on therapy effectively reduces proteinuria in chronic kidney disease: an open-label prospective trial. J Renin Angiotensin Aldosterone Syst.

[57] Dieterle W, Corynen S, Mann J (2004). Effect of the oral renin inhibitor aliskiren on the pharmacokinetics and pharmacodynamics of a single dose of warfarin in healthy subjects. Br J Clin Pharmacol.

[58] Chang J, Yang W, Kahler KH, Fellers T, Orloff J, Bensimon AG, Yu AP, Fan CP, Wu EQ (2011). Compliance, persistence, healthcare resource use, and treatment costs associated with aliskiren plus ARB versus ACE inhibitor plus ARB combination therapy: in US patients with hypertension. Am J Cardiovasc Drugs.

[59] Vaidyanathan S, Reynolds C, Yeh CM, Bizot MN, Dieterich HA, Howard D, Dole WP (2007). Pharmacokinetics, safety, and tolerability of the novel oral direct renin inhibitor aliskiren in elderly healthy subjects. J Clin Pharmacol.

[60] Fischer MA, Avorn J (2004). Economic implications of evidence-based prescribing for hypertension: can better care cost less?. JAMA.

[61] Delea TE, Sofrygin O, Palmer JL, Lau H, Munk VC, Sung J, Charney A, Parving HH, Sullivan SD (2009). Cost-effectiveness of aliskiren in type 2 diabetes, hypertension, and albuminuria. J Am Soc Nephrol.

